# Measuring stress in Australia: validation of the perceived stress scale (PSS-14) in a national sample

**DOI:** 10.1186/s12955-020-01343-x

**Published:** 2020-04-15

**Authors:** Pedro H. Ribeiro Santiago, Tine Nielsen, Lisa Gaye Smithers, Rachel Roberts, Lisa Jamieson

**Affiliations:** 1 Adelaide Dental School, The University of Adelaide, AHMS Building, North Terrace, Adelaide, SA 5000 Australia; 2 Department of Psychology, The University of Copenhagen, Copenhagen, Denmark; 3 School of Public Health, The University of Adelaide, Adelaide, Australia; 4 School of Psychology, The University of Adelaide, Adelaide, Australia; 5 Adelaide Dental School, The University of Adelaide, Adelaide, Australia

**Keywords:** Psychometrics, Perceived stress scale, Australia, Differential item functioning, Measurement invariance, Psychological stress, Rasch analysis

## Abstract

**Background:**

In Australia, the stress levels have increased over the years, impacting on the physical and mental health of the general population. The aim of the present study was to evaluate the validity and reliability of the PSS-14 in an Australian population.

**Methods:**

The PSS-14 was applied to a large national sample comprising 3857 Australians in the population-based cross-sectional study Australia’s National Survey of Adult Oral Health 2004–2006. The psychometric properties analyzed with the Rasch model and Graphical Log-linear Rasch models were: model fit, item fit, local dependence, differential item functioning, unidimensionality, reliability, targeting and criterion validity.

**Results:**

The PSS-14 did not fit the pure RM (χ2 (55) = 3828.3, *p* = < 0.001) and the unidimensionality of the whole scale was rejected (*p* = < 0.001). The Perceived Stress (χ2 (27) = 1409.7, *p* = < 0.001) and Perceived Control (χ2 (27) = 713.4, *p* = < 0.001) subscales did not fit the pure RM. After the deletion of two items, the Perceived Stress subscale (χ2 (96) = 94.4, *p* = 0.440) fitted a GLLRM, while the Perceived Control scale (χ2 (55) = 62.50, *p* = 0.224) fitted a GLLRM after the exclusion of four misfitting items.

**Conclusions:**

The Perceived Stress subscale displayed adequate psychometric properties after the deletion of two items; however, the majority of problems centered around the Perceived Control subscale. The presence of differential item functioning among four items indicates that adjustment of total scores is required to avoid measurement bias. Recommendations for future applications in Australia are provided.

## Introduction

In Australia, the Australian Psychological Society (APS) conducted a ‘State-of-the-Nation’ Stress & Well-Being Survey (SWBS) from 2011 to 2015 to investigate stress at a national level [1, 2]. The results showed that almost two in three Australians (64%) reported that stress was impacting their mental health, while approximately one in five (17%) reported that stress was strongly impacting their physical health [3]. The findings from the last survey, which had 1731 respondents, indicated that compared to 2011 the levels of stress increased, and the levels of well-being decreased in the Australian population. One of the concerning findings was that, among those with severe levels of distress, 61% drank alcohol, 41% gambled, 40% smoked and 31% used recreational drugs as a coping mechanism [2]. The surveys also revealed gender differences. Women were consistently more affected by stress than men and reported financial and health issues as their main sources of concern [3].

One of the many psychological instruments used in the SWBS was the Perceived Stress Scale (PSS) [4]. The PSS is the world’s most widely used instrument to measure perceived stress [5] and since its development has been continuously applied in empirical research [6, 7]. The PSS was developed based on the theoretical perspective of Lazarus [8], which rather than focusing on external environmental stressors, postulated that the stress response is determined by the *perception* of these environmental stressors. According to Lazarus [8], life events, such as divorce or losing a job, only cause stress when they are *appraised as threatening (*e.g. “I don’t have another job”*)* and there is a *perception of insufficient coping resources (*e.g. “I don’t know anyone who could employ me”). The measurement of stress has then been operationalized in two ways: the *environmental perspective* (e.g. using life-event scales) and the *psychological perspective* (e.g. using perceived stress scales) [9, 10]. The PSS was developed to measure stress from the *psychological perspective*, diverging from the life-event scales regularly used at that time [11]. The initial validations conducted by Cohen [4, 12] led to the creation of two shortened scales derived from the original 14 item-version (PSS-14): the PSS-10 and the PSS-4.

The results of the SWBW surveys were nationally reported by the Australian media (see “Australian women feel more stressed than men, mental health survey finds” [13]). However, the reports did not specify which PSS version was used and indicated only that the “level of stress was derived by summing the scores of the 11 scale items” [2]. Additionally, evidence of validity was not provided. Considering the high levels of stress reported in the Australian population, it is necessary to ensure that psychological measures applied to measure stress in Australians are valid and reliable, so it is possible to have confidence in the interpretation of test results. In the present study, we aim to investigate the psychometric properties of the PSS-14 in the general Australian population and examine whether this instrument can provide a valid measure of perceived stress for future research. To evaluate the PSS-14 validity and reliability we used data collected for the Australia’s National Survey of Adult Oral Health (NSAOH) 2004–2006, a broad project originally aimed to determine the psychosocial determinants of oral health in Australia. Despite being conducted prior to the SWBW, the NSAOH 2004–2006 has a large national sample (*n* = 3857) that can provide evidence of the PSS-14 validity in the Australian general population.

### The present research

The psychometric properties of the PSS have been evaluated in multiple countries [14]. There are, however, two main limitations regarding the generalizability of its psychometric properties to an Australian population. Firstly, the majority of studies evaluated the PSS-14 in small and/or non-representative samples [14]. For example, in China, the PSS-14 was evaluated in a sample of 1860 cardiac patients who smoked [15], while the PSS-10 was evaluated in a sample of policewomen [16]. Secondly, other studies were conducted in countries culturally and economically diverse from Australia, such as the application of the PSS-10 to 479 adults in Thailand [17], a country known for its “collectivist Eastern culture” [18]; or the application of the PSS-14 to 941 adults in Greece [19], which recently experienced financial crisis [20]. Among all countries studied, Canada is the western developed nation most similar to Australia due to its “large geography, low population density and similar health care challenges” [21]. However, the PSS-14 was initially applied in Canada to 96 psychiatric patients [22] and the PSS-4 was later evaluated in 217 pregnant women [23]. The peculiarity of the samples from Canada (i.e. psychiatric patients) and most countries in general makes it difficult to generalize the results to typical members of the Australian general population. For the most part, the PSS has been validated in samples experiencing stressful environments (i.e. patients, students, policemen) rather than in general populations [14].

The most relevant study in a population similar to Australia continues to be the validation conducted by Cohen and Williamson [12] in a representative sample of 2387 Americans. Both countries, Australia and United States (US), are large high income countries [24], with a history of English colonization [25] and populations with similar demographic characteristics [26] and morbidity patterns [27, 28]. Nevertheless, there are important dissimilarities in terms of social-political context between these countries. For example, in the US, the national health system is a private employer-based and individual insurance program that provides coverage to 90% of the population, while Australia has a universal public insurance program covering 100% of the individuals [26]. Although finances are the main source of stress both in Australia [2] and the US [29], these are structural differences regarding how these environmental stressors are experienced by each population (i.e. concerns with *health* costs are more prominent in the US).

One important characteristic of the Australian population is the cultural background of its Indigenous groups, namely Aboriginal Australians and Torres Strait Islanders (ABTSI). The Aboriginal Australians experiences of well-being are rather distinct from western individuals [30] and “Western psychological concepts are inappropriate and potentially damaging to Indigenous people” [31]. One example is the PSS-14, which was recently validated for an Aboriginal population and the findings showed a weak latent correlation between the “Perceived Stress” and “Perceived Coping” subscales (*r* = 0.14), a result distinct from the moderate (*r* = 0.50) to strong (0.70) correlations found in western societies [32]. For these reasons, we followed the recent recommendations by Kowal, Gunthorpe [31] and Santiago, Roberts [32] that ABTSI are a culturally distinct group in which psychological instruments should be evaluated separately from the general Australian population.

Hence, the present study aims to (1) investigate the psychometric properties of the PSS-14 in the general Australian population. We hypothesize that the functioning of the PSS-14 in the Australian population is similar but not equal to its functioning in other high-income countries. In addition, we aim to (2) updated the evidence about the PSS-14 functioning in developed countries using a large national sample and (3) further advance the knowledge regarding the PSS psychometric properties using item-response theory to investigate issues of differential item functioning (DIF) and local dependence (LD). The previous research about stress in Australia showed that “Australian women feel more stressed than men” [13]. Although this result is common in many western countries, a long-established questioning is whether those differences are due to measurement bias [14, 33]. Therefore, we aim to (4) investigate gender difference in PSS scores, and whether differences were due to measurement bias.

Finally, we aim to evaluate criterion validity by inspecting convergence and divergent validity with two psychological constructs (social support and stress at work) of the perceived stress’ nomological network [34]. Social support has been shown by a large body of research as a protective (or *buffering)* factor against stress [35]. Social support refers to the functions performed by family, friends, and significant others when an individual encounters an external environmental stressor [36]. In this case, family, friends or significant others can help to change the situation (e.g. helping with a task at work) or change *the meaning* of the situation (e.g. help interpreting the event from a less distressing or extreme perspective) [37]. In both cases, the individual has additional resources to deal with the *environmental stressor* and this decreases his *perception* of how stressful the situation is [38].

On the other hand, psychological stress can be experience at work due to a demanding environment. One theoretical model that explains how the work environment generates stressful experiences is the *effort-reward imbalance* [39]. The model indicates that when the rewards received at work did not correspond to the efforts employed (‘high cost/low gain’), the imbalance can lead adverse stress responses [40]. Therefore, it is expected that participants with high perceived stress will have low social support from friends, family and significant others and experience more efforts with less rewards at work.

To achieve these aims, we analysed data from Australia’s National Survey of Adult Oral Health (NSAOH) 2004–2006, a broad project originally designed to determine the psychosocial determinants of oral health in the Australian population. The NSAOH was chosen since it provides the best available data for the evaluation of the PSS-14 validity in the Australian population. Firstly, the NSAOH sample comprises the largest national Australian sample (*n* = 3857) in which the PSS-14 has been applied. Secondly, the NSAOH achieved high standards of response quality for surveys [41], including high response rates (77.4%) [42] and low missingness of individual items (0.0 to 1.3%). Survey response rates have declined over the decades, with average rates below 50% been consistently reported since the 1990s [43]. In summary, the large sample recruited at a national level and the high-quality PSS-14 item responses qualified the NSAOH as the preferred data for our research question.

## Methods

### Participants and procedures

The sample comprised 3857 non-Aboriginal Australians in the population-based cross-sectional study Australia’s National Survey of Adult Oral Health 2004–2006. The NSAOH 2004–2006 was a broad project aimed to determine the psychosocial determinants of oral health in Australia. The survey had a three-stage (i.e. postcodes, households, people) stratified clustered sampling design to select a representative sample of Australian adults. Participants were contacted by study staff who conducted a computer-assisted telephone interview. Interviewees that agreed to undertake dental examinations were mailed the PSS-14 (Supplementary Table 1 – Additional file 1), along with the other complementary measures, as part of a larger questionnaire. The NSAOH 2004–2006 was approved by the University of Adelaide’s Human Research Ethics Committee. All participants provided signed informed consent [44]. A sample of 42 Aboriginal Australians was removed from the analysis since the PSS-14 has been previously validated for this group [32] and it is recommended that psychometric research with Indigenous groups should be conducted separately due to cultural differences [31].

### Psychometric properties of the perceived stress scale

The psychometric properties of the PSS have been evaluated in multiple countries, including Spain, Canada, Brazil, Ethiopia and Japan, and its most studied property is dimensionality. There is a consensus, mostly from factor analytical studies, that the PSS has a two-dimensional structure, composed of negatively worded and positively worded items [14]. These two dimensions are consistent with Lazarus’s [8] theory and were named the “Perceived Stress” and “Perceived Control” subscales, although other terminologies such as “Perceived Distress” and “Perceived Coping” have also been used [22].

Considering the robust evidence regarding dimensionality, a few psychometric studies have started to evaluate DIF. One main hypothesis analysed is if the PSS items are biased according to gender [5, 33, 45–48]. Since women have consistently scored higher than men in the Perceived Stress subscale (but not on the Perceived Control subscale [22, 33, 47], a long-lasting debate in the PSS literature is if score differences are “an artifact of measurement bias” or “true gender differences arising from social, biological, or psychological influences” [14]. The findings regarding DIF by gender are mixed [5, 33, 45–49]. Although some studies indicated no evidence of DIF [5, 33, 46], Cole [45] reported that PSS-10 items 3, 6, 7, 8 and 10 had DIF with a small magnitude and suggested that the “combination of the potentially slightly biased items may explain the apparent test level bias towards women”. Gitchel et al. [47] found DIF by gender for PSS-10 items 1, 3, 4 and 6, a result partially confirmed by Nielsen and Dammeyer [48] (i.e. which also reported DIF for Items 1 and 3). Other sources of DIF have also been investigated. Regarding education, DIF was found for the PSS-10 items 3, 4, 8 and 9 [45], while other studies analyzed age, ethnicity, and literacy [45, 49].

The analysis of LD of PSS items has only recently started [48, 50]. The investigation of LD is especially relevant for the PSS since, in many of the PSS-14 studies, the two-factor structure accounted for less than 50% of the total variance [14]. These findings suggest that a high percentage of the variance of item responses is not explained by the latent trait, and the PSS literature is still not clear regarding what these other influences could be.

Finally, the PSS-14 has previously displayed adequate reliability in different samples. The internal consistency reliability, measured by the Cronbach’s α [51], was higher than .70 in 11 of 12 studies, while the test-retest reliability was higher than .70 in 2 of 3 studies [14]. However, since Cronbach’s α provides a lower-bound estimate of reliability when items are locally independent [52], the analysis of LD of PSS items is required to ensure that reliability estimates are not inflated [50].

### Complementary measures

#### The perceived stress scale (PSS)

The PSS is a five-point scale (1 = Strongly Disagree, 2 = Disagree, 3 = Neutral, 4 = Agree, 5 = Strongly Agree) with a two-factor structure of perceived Stress (PS) and perceived Coping (PC) which evaluates if a person’s life is perceived as unpredictable, uncontrollable, or overloading [4].

The two complementary measures used in this study in the analysis of criterion validity were:

- *The Multidimensional Scale of Perceived Social Support (MSPSS):* The MSPSS is a 12 item five-point scale (1 = Strongly Disagree, 2 = Disagree, 3 = Neutral, 4 = Agree, 5 = Strongly Agree), with a three-factor structure of family (FA), friend (FR) and significant others (SO) [53]. The MSPSS containing all 12 items (α = 0.93) and the FA (α = 0.92), FR (α = 0.92) and SO (α = 0.95) subscales displayed excellent reliability.
- *The Efforts-Reward Imbalance Questionnaire (ERI):* A shorter version of the five-point scale (1 = Strongly Disagree, 2 = Disagree, 3 = Neutral, 4 = Agree, 5 = Strongly Agree) ERI questionnaire with 11 items was used. The ERI questionnaire has a three-factor structure composed of effort (EF), reward (RD) and over commitment (OC) [40]. The ERI containing all 11 items (α = 0.75) and the ER (α = 0.85) and RD (α = 0.73) subscales displayed adequate reliability. The OC (α = 0.52) subscale displayed poor reliability and for this reason was not included in the analysis of criterion validity.

### The Rasch measurement models

The Rasch model (RM) is part of the family of Item Response Theory (IRT) models and it has two distinctive features over other IRT models: (1) the sum score is a sufficient statistic for the person parameter, containing all the information that allows statistical inference about the latent trait [54]; and (2) inference can be conducted on a conditional framework [55], since person and item parameters can be eliminated by means of conditional probabilities [56], a property that Rasch [57] referred as *specific objectivity*.

A mathematical property of the RM is the conditional independence of item responses to exogenous variables (i.e. absence of DIF) and to other items (i.e. local independence). However, in most rating scales applied in health sciences, items often show evidence of LD and DIF. Therefore, items with LD or DIF do not fit the RM [58] and a common practice has been the deletion of items solely to obtain statistical fit to the model [59, 60]. This practice is problematic; if the deleted items cover important aspects of the construct, there is a threat to content validity [61] that can lead to “construct underrepresentation” [62]. In addition, the revised scale might end up being composed of a small number of items, leading to reduced reliability [58].

For this reason, recent methodological advances consist of analysis by Graphical Loglinear Rasch Model (GLLRM), which extends the RM with additional parameters to incorporate uniform LD and uniform DIF [60]. The term *uniform* refers to when the magnitude of the conditional dependence between items (LD) or between an item and an exogenous variable (DIF) is constant across the trait level. GLLRM is a combination of two independently developed statistical methods. The first method is the log-liner IRT models developed by Kelderman [63, 64], which generalizes IRT models to relax the assumption of local independence. The assumption of local independence is restrictive and frequently not achieved by questionnaires in health sciences. Therefore, log-liner IRT models allows locally dependent items, while representing traditional IRT models with locally independent items (e.g. Partial Credit model) as a special case. The second method is the development of Graphical models [65], which graphically represent the structure of conditional dependence between variables. Since in the RM the total score is a sufficient statistic for the person parameter, graphical models are suitable for the analysis of LD and DIF. For example, to evaluate DIF, items and exogenous variables should be conditionally independent given the total score. The structure of conditional dependence between items, latent trait and exogenous variables can then be represented graphically.

The functional form of a general GLLRM (containing one LD and one DIF parameter) can be expressed as:

$$ \ln \Big(P\left(Y=\left({y}_1,\dots, {y}_k\Big)|\theta, C\ \right)\right)={\lambda}_0\left(\theta, x\right)+\sum \limits_i\left({\alpha}_{y_i}^i+{y}_i\theta \right)+\sum \limits_{i,j}{\lambda}_{y_i{y}_j}^{i,j}+\kern0.5em \sum \limits_{i,j}{\delta}_{y_i{c}_j}^{i,j}\kern0.5em $$

which describes the conditional distribution of a vector of item responses (y_1_, …,y_k_) given the latent trait θ and exogenous variables C. The terms ${\lambda}_0\left(\theta, x\right)+\sum \limits_i\left({\alpha}_{y_i}^i+{y}_i\theta \right)$ are equivalent to the RM for polytomous items (i.e. Partial Credit model), while ${\lambda}_{y_i{y}_j}^{i,j}$ represents the interaction parameter between *item i* and *item j* and ${\delta}_{y_i{c}_j}^{i,j}$ represents the interaction parameter between *item i* and *exogenous variable j*. For an in-depth technical discussion of GLLRMs, please see [59].

The usefulness of GLLRM is that, when questionaries exhibit uniform LD and uniform DIF, departures from the RM do not necessarily imply that items are flawed: locally dependent items convey less information than independent items and lead to reduced reliability; items with DIF require scores to be adjusted to allow comparison between subgroups. However, in both cases, the item serves its original purpose of measuring the latent trait, and retaining these items is important to preserve construct validity. Furthermore, in both cases, the distinctive feature of the RM is preserved: if the uniform LD parameter is included the sufficiency of the total score is retained; while, if the uniform DIF parameter is present, the sufficiency of the total score is retained within the DIF-defined subgroups [59]. Finally, the uniform LD and DIF parameters can inform how items deviated from ideal measurement requirements and become a starting point for modifications on an instrument level [58]. This approach aims to investigate *why* items did not fit the RM; and when departures consist of uniform LD and uniform DIF, it is possible to retain the items and inform future modifications on the instrument [58].

### Statistical analysis

#### Item analysis

Item analysis was conducted with the following steps: (1) initially testing if the items would fit the RM [66]; (2) if fit to the RM was rejected, the departures were investigated and catalogued; and (3) in case of uniform LD and uniform DIF, the fit to a GLLRM adjusting for these departures was tested. In case of other types of departures, such as items displaying evidence of being a poor measure of the construct, the most problematic item was removed and the three previous steps repeated. The estimation method for the RM and GLLRM was conditional maximum likelihood [55]. Person parameters were estimated using weighted maximum likelihood (WML) [67]. Since missing values for individual items ranged from 0.0 to 1.3%, multiple imputation was not required [68]. All statistical analyses were conducted with the DIGRAM v4.05 [69, 70]. Descriptive statistics and graphs were created with R software [71]. The item analysis included the evaluation of: a) model fit; b) global DIF; c) item fit; d) LD; e) DIF; and f) unidimensionality. After a measurement model was established,: g) reliability and h) targeting of the instrument in this sample was evaluated.

#### Model fit and global DIF

Overall fit of the model was evaluated through the Conditional Likelihood Ratio (CLR) test [72]. The CLR test evaluates if item parameters are *invariant* between subsamples. One distinctive feature of items fitting a RM (and GLLRMs, see [59]) is that, within a specific frame of reference (e.g. Australian general population) [57], the functioning of the instrument (e.g. the difficulty of the items) is independent of the sample in which the instrument has been applied. Hence, if items do fit a RM/GLLRM, it is possible to divide the study sample according to a chosen criteria (i.e. lower and higher scores) and item parameters should remain the same in both subsamples. For this reason, the CLR test is a fit statistic to evaluate overall fit to the RM [69]. Moreover, when the sample is divided according to criteria based on exogenous variables (e.g. smokers/non-smokers, men/women) and item parameters were found not to be invariant, the CLR test provides evidence of (Global) DIF. In our study, the subsamples were defined according to lower and higher scores (i.e. homogeneity) to evaluate overall model fit; and according to sex (Male; Female) and education (education level up to High School; Technical education^1^ or University) to evaluate Global DIF [54].

#### Item fit

The investigation of fit at an item level evaluates whether the observed responses to a specific item are in accordance with the responses predicted by the RM/GLLRM model. Fit of individual items was evaluated by conditional infit and outfit statistics, which, differently from traditional infit and outfit statistics, have a known sampling distribution [74].

#### LD and DIF

To investigate LD and/or DIF, Kelderman’s [64] likelihood ratio (LR) test was conducted to test if the additional uniform LD or uniform DIF parameter would better explain the item responses compared to the fitted model. In addition, the *magnitude* of the uniform LD or uniform^2^ DIF was evaluated through the partial Goodman-Kruskal [75] *γ* rank correlation between items given the two restscores or between item and exogenous variable given the total score [76]. In case DIF was present, the scores were adjusted and conversion tables reported [59]. When multiple tests were performed, the Benjamini-Hochberg [77] procedure was conducted to adjust for false discovery rate (FDR).

#### Dimensionality

Initially, the RM and subsequent GLLRMs were tested for the PSS-14 containing all items. In case no fit was found, we then proceeded to test the two subscales composed of negatively and positively worded items. Finally, if a RM or GLLRM was found for each subscale, a formal test of unidimensionality was conducted by comparing the observed *γ* correlation of the subscales with the expected *γ* correlation of the subscales under the unidimensional model. The rationale is that the correlation between two subscales measuring different traits is weaker than the expected correlation of subscales measuring the same trait [78]. Negatively worded items (from the “Perceived Stress” subscale) were reverse scored in the dimensionality analysis. Markov graphs [59] were reported to illustrate the final models.

#### Reliability

In case of fit to the RM, reliability was estimated using Cronbach’s *α* [51], since it provides a lower-bound estimate of reliability [52] when items are locally independent. However, when LD was found, a Monte Carlo simulation method [79] that adjusts for the LD between items was applied. Since DIF implies that the *item thresholds* (and, consequently, the *item difficulty*) change according to subgroup, the different item parameters influence the true score distribution so reliability was calculated for each subgroup independently [80]. In addition, the person separation probability was calculated, which is the probability that the total scores rank two random persons in the same way as the *true* value of their latent trait (i.e. rather than the *estimates*).

#### Targeting

Targeting was evaluated through the Test Target Information Index, which consists of the mean test information divided by the maximum obtained test information. In addition, targeting was evaluated graphically through the inspection of item maps.

#### Criterion validity

Since scores are ordinal, the convergent and divergent validity of the PSS with other psychological constructs pertaining to its nomological network [34] was evaluated by calculating the non-parametric Kendall’s τ [81]. For this analysis, the complementary measures were used. A negative correlation of Perceived Stress with FA, FR, SO and RW, and a positive correlation with EF and OC was anticipated. In addition, known-groups validity [82] was assessed and it was expected that women would have higher scores on the Perceived Stress subscale [14] but no difference in scores on the Perceived Control subscale [22, 33, 47]. It was also expected that participants with less education would have higher scores on the Perceived Stress subscale [14].

## Results

The demographic characteristics of the sample are found in Table 1. Participants age ranged from 18 to 82 years (M = 50.2, SD = 14.8). The majority of participants were women (61.9%), had a tertiary education (67.5%) and were employed (59%).

**Table 1:** Characteristic of the study participants

	n	%
Age		
Mean	50.3	
SD	14.8	
Min/Max	18–82	
Missing	0	0%
Sex		
Female	2388	61.9%
Male	1469	38.1%
Missing	0	0%
Education		
High school or less	1252	32.5%
Technical education or university	2605	67.5%
Missing	0	0%
Employed		
Yes	2274	59%
No	1583	41%
Missing	0	0

Mean values, minimum, maximum and standard deviations; numbers and percentages

### PSS-14

Fit of the PSS-14 to the RM was rejected (Table 2).

**Table 2:** Conditional likelihood ratio test of overall model fit and Global DIF

	Model	Homogeneity	Differential Item Functioning by sex	Differential Item Functioning by education
PSS-14	RM	χ^2^(55) = 3828.3, *p* < 0.001	χ^2^(55) = 575.1, *p* < 0.001	χ^2^(55) = 320.9, *p* < 0.001
Perceived Stress	RM	χ^2^(27) = 1409.7, *p* < 0.001	χ^2^(27) = 177.8, *p* < 0.001	χ^2^(27) = 82.2, *p* < 0.001
	GLLRM	χ^2^(96) = 94.4, *p* = 0.440	χ^2^(80) = 111.8, *p* = 0.012	χ^2^(88) = 104.1, *p* = 0.080
Perceived Control	RM	χ^2^(27) = 713.4, *p* < 0.001	χ^2^(27) = 197.2, *p* < 0.001	χ^2^(27) = 104.1, *p* < 0.001
	GLLRM	χ^2^(55) = 62.5, *p* = 0.224	χ^2^(39) = 39.0, *p* = 0.469	χ^2^(47) = 70.9, *p* = 0.014

The subgroups were defined according to lower and higher scores (i.e. homogeneity) to evaluate overall model fit; and according to sex (men; women) and education (Up to high school; Technical education or University) to evaluate Global Differential Item Functioning

The results indicated item misfit (Supplementary Table 2 - Additional file 1) among the majority of items. The analysis proceeded by sequentially excluding items, such as items 4, 5, 9, 12, 13, and 6 that displayed the highest misfit, while investigating departures in terms of LD and DIF with GLLRMs. However, it became clear that: a) LD and DIF could not explain the misfit to the RM and GLLRMs were not found; and b) the majority of excluded items were negatively worded, indicating that they would not form a unidimensional scale together with the positively worded items. At this point, we proceeded to the analysis of the subscales.

### Perceived stress subscale

**Fig. 1:**
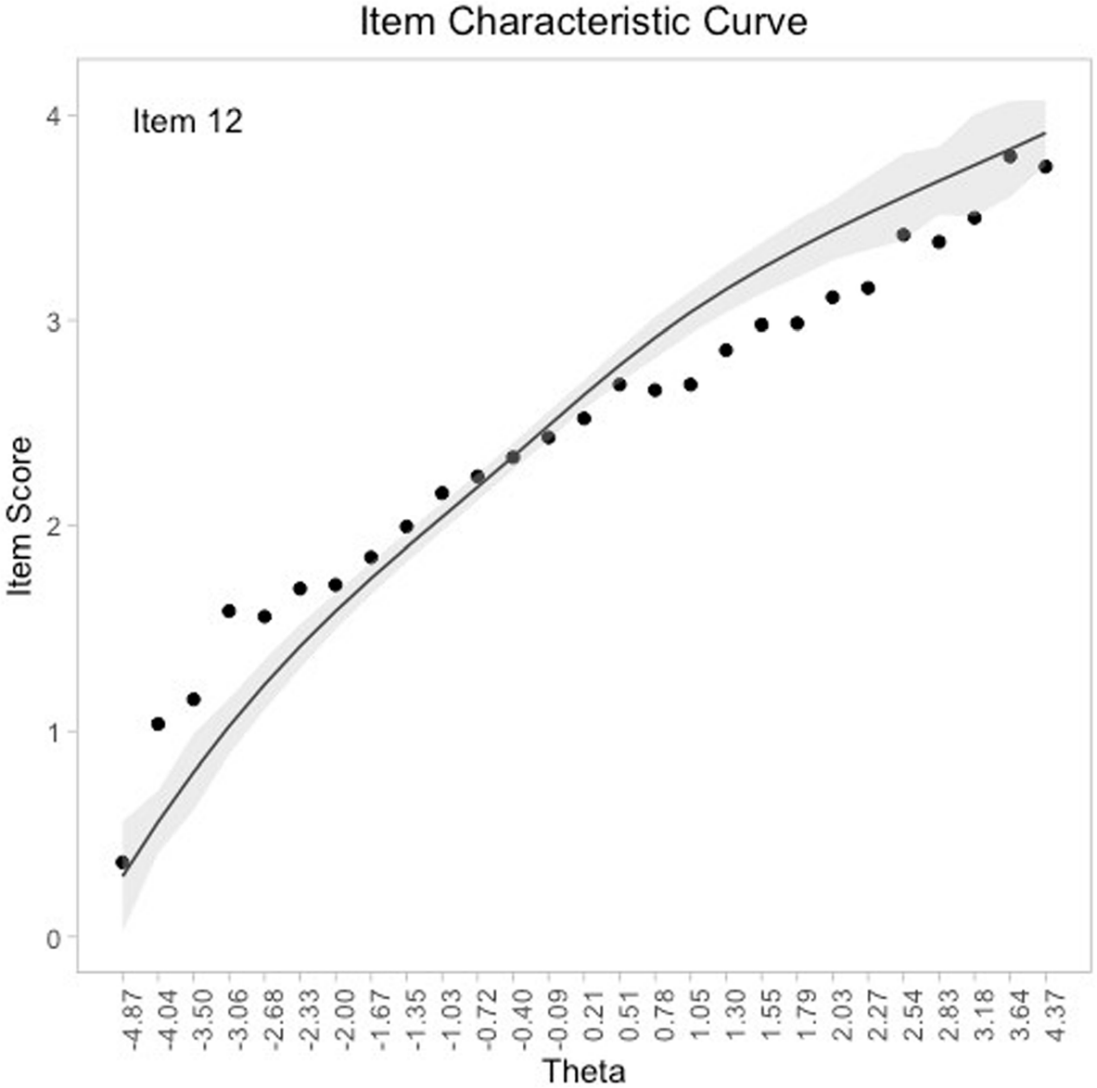
Item characteristic curve for Item 12. Note. The x-axis indicates the latent trait and the y-axis indicates the item score. The black points represent the observed item responses for each total score. The grey curve is the expected item responses and the grey shaded area is the 95% confidence regions

Fit of the negatively worded items (“Perceived Stress”) subscale to the RM was rejected (Table 2). The investigation of item fit statistics (Supplementary Table 3 - Additional file 1) indicated strong misfit of Item 12 ( “… found yourself thinking about all the things you have to accomplish?”) (Infit = 1.675, SE = 0.023, *p* < 0.001; Outfit = 1.669, SE = 0.023, *p* < 0.001) (Fig. 1).

Figure 1 shows that the average observed scores exhibited a pattern of *under discrimination* since they formed a flat curve compared to the model expectations, indicating that item responses were less influenced by the latent trait (“perceived stress”). It was then evaluated whether Item 12 misfit could be a result of DIF or LD (i.e. although LD often results in *over discrimination*) but a GLLRM was not found. For these reasons, Item 12 was excluded.

After the deletion of Item 12, the CLR test rejected fit to the RM (χ^2^ (23) = 312.9, *p* < 0.001) and the next item that displayed misfit was Item 8 ( “… felt unable to cope with all the things that you had to do?”) (Infit = 1.145, SE = 0.023, *p* < 0.001; Outfit = 1.155, SE = 0.023, *p* < 0.001). The analysis indicated that Item 8 misfit was also not a result of LD or DIF and Item 8 was also excluded.

### GLLRM of the perceived stress subscale

**Fig. 2:**
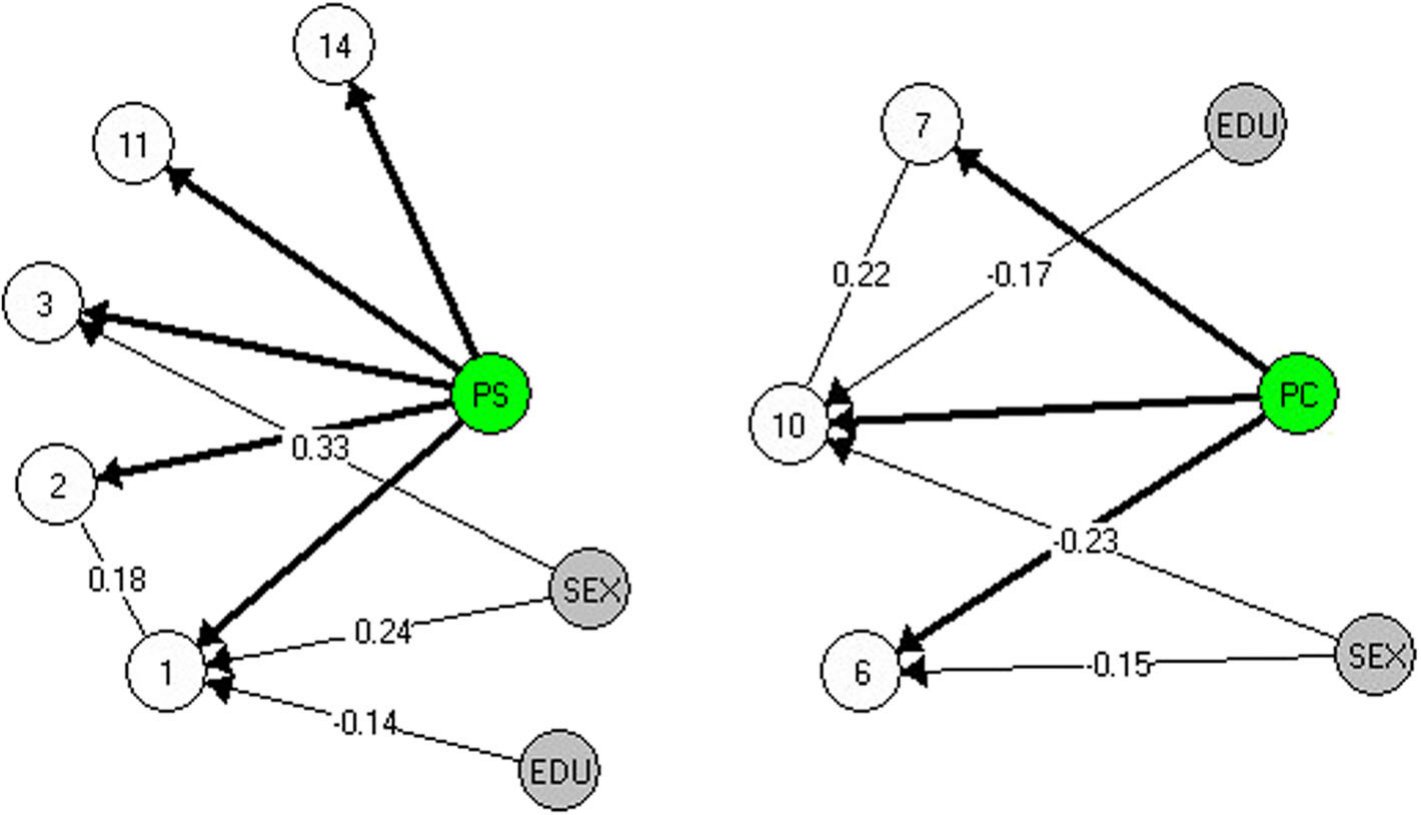
GLLRMs of the Perceived Stress subscale (left) and Perceived Control subscale (right). Note. The Markov graph nodes represent the item numbers, the exogenous variables and the latent trait. Disconnected nodes indicate that variables are conditionally independent and partial *γ* informs the magnitude of the local dependence and differential item functioning

After exclusion of the two items, the CLR test rejected fit to the RM but fit to a GLLRM was found (χ^2^ (96) = 94.4, *p* = 0.440) (Table 2) (Fig. 2).

LD was found between Item 1 ( “… felt upset because of something that happened unexpectedly?”) and Item 2 ( “… felt unable to control the important things in your life?”) (γ_avg_ = 0.18). DIF was found between Item 1 and sex (γ = 0.24); between Item 3 ( “… felt either nervous or stressed?”) and sex (γ = 0.33); and between Item 1 and education (γ = − 0.14). There was no item misfit (Table 3), and the Kelderman’s LR test indicated no further evidence of DIF or LD (Supplementary Table 4 - Additional file 1).

**Table 3:** Item fit statistics for the GLLRM of the negatively worded items (“Perceived Stress”)

Item	Conditional Outfit			Conditional Infit		
	Observed	SE	*p*-value	Observed	SE	*p*-value
Item 1	1.021	0.029	0.482	1.024	0.028	0.386
Item 2	0.950	0.031	0.108	0.948	0.026	0.049
Item 3	0.993	0.027	0.783	0.991	0.025	0.726
Item 11	1.015	0.026	0.550	1.024	0.025	0.355
Item 14	0.991	0.024	0.702	0.994	0.024	0.806

The Conditional Outfit and Conditional Infit statistics have expected values equal to one under the Rasch model

Considering that the GLLRM had overall model fit and there was no further evidence of global DIF, item misfit, DIF or LD, the measurement model for the “Perceived Stress” subscale was established.

### Perceived control subscale

Fit of the positively worded items (“Perceived Control”) subscale to the RM was rejected (Table 2). Misfit was found among the majority of items (Supplementary Table 5 - Additional file 1). The item with the highest misfit was Item 9 ( “… felt able to control irritations in your life?”) (Infit = 1.367, SE = 0.026, *p* < 0.001; Outfit = 1.237, SE = 0.023, *p* < 0.001) and it was excluded. On the subsequent analysis, substantial misfit was also found regarding Item 13 ( “… felt able to control the way you spend your time?”) (Infit = 1.363, SE = 0.036, *p* < 0.001; Outfit = 1.180, SE = 0.032, *p* < 0.001), Item 4 ( “… dealt successfully with irritating life hassles?”) (Infit = 1.226, SE = 0.024, *p* < 0.001; Outfit = 1.185, SE = 0.024, *p* < 0.001) and Item 5 (“...effectively coped with important changes in your life?”) (Infit = 1.571, SE = 0.024, *p* < 0.001; Outfit = 1.501, SE = 0.024, *p* < 0.001) and these items were removed.

### GLLRM of the perceived control subscale

After the exclusion of the misfitting items, the CLR test indicates fit to a GLLRM (χ^2^ (55) = 62.5, *p* = 0.224) (Table 2) (Fig. 2). LD was found between Item 7 ( “… felt things were going your way?”) and Item 10 ( “… felt you were on top of things?”) (γ_avg_ = 0.22). DIF was found between Item 10 and sex (γ = − 0.23); between Item 6 (“...felt confident about your ability to handle your personal problems?”) and sex (γ = − 0.15); and between Item 10 and education (γ = − 0.17). There were no further evidence of item misfit (Supplementary Table 6 - Additional file 1) or LD/DIF (Supplementary Table 7 - Additional file 1). Considering that the GLLRM had overall model fit and there was no further evidence of global DIF, item misfit, LD or DIF, the measurement model for the “Perceived Control” subscale was established.

The table for adjusting scores after accounting for DIF is provided for both subscales (Supplementary Table 8 - Additional file 1).

### Dimensionality

Since the observed correlation between the Perceived Stress and Perceived Control subscales (*γ*= 0.527) was weaker than the expected correlation between the two subscales (*γ* = 0.569, SE = 0.009, *p* < 0.001) under a unidimensional model, the unidimensionality of the PSS-14 was rejected. Therefore, unidimensionality was confirmed *within* subscales but not *between* subscales, indicating that the Perceived Stress subscale and the Perceived Control subscale measure qualitatively distinct psychological traits.

### Targeting and reliability

For the Perceived Stress subscale, the targeting was moderate. The overall Test Information Target Index indicates that for the Australian population the Perceived Stress subscale provided only 60% of the total information available if the instrument was perfectly targeted. Values ranged from 56 to 62% within subgroups (Table 4). For example, women who completed Technical education or University had an average total score of 8.48 (SD = 3.65), while the Perceived Stress subscale was perfectly targeted for a population with an average score of 14.79 (SE = 1.97). The overall reliability was 0.84. The overall person separation probability was 83%, indicating that if two respondents were randomly selected and then ranked on their total score, in 83% of cases they will be ranked correctly according to their true level of perceived stress.

**Table 4:** Targeting and reliability information of the Perceived Stress and Perceived Control subscales

Subgroup			Score			Target Index	Reliability	Probability of Person Separation
Education	Sex	n	Mean	SD	Target			
Perceived Stress subscale								
Up to High School	Male	392	7.51	3.99	14.83	0.56	0.85	0.83
Technical education or Uni	Male	1075	7.41	3.70	14.85	0.58	0.83	0.82
Up to High School	Female	858	8.53	4.02	14.79	0.60	0.86	0.84
Technical education or Uni	Female	1525	8.48	3.65	14.79	0.62	0.82	0.82
Perceived Control Subscale								
Up to High School	Male	392	4.29	2.45	9.18	0.36	0.77	0.75
Technical education or Uni	Male	1070	3.72	2.18	9.07	0.34	0.73	0.74
Up to High School	Female	857	4.14	2.20	9.28	0.34	0.75	0.75
Technical education or Uni	Female	1526	3.91	2.12	9.20	0.34	0.71	0.73

The mean score is the average score for each subgroup. The target is the score which maximizes the information function. Reliability is the proportion of true score variance in relation to the total score variance. The probability of person separation is the probability that the scores of two random persons have the same rank order as their true person parameters

**Fig. 3:**
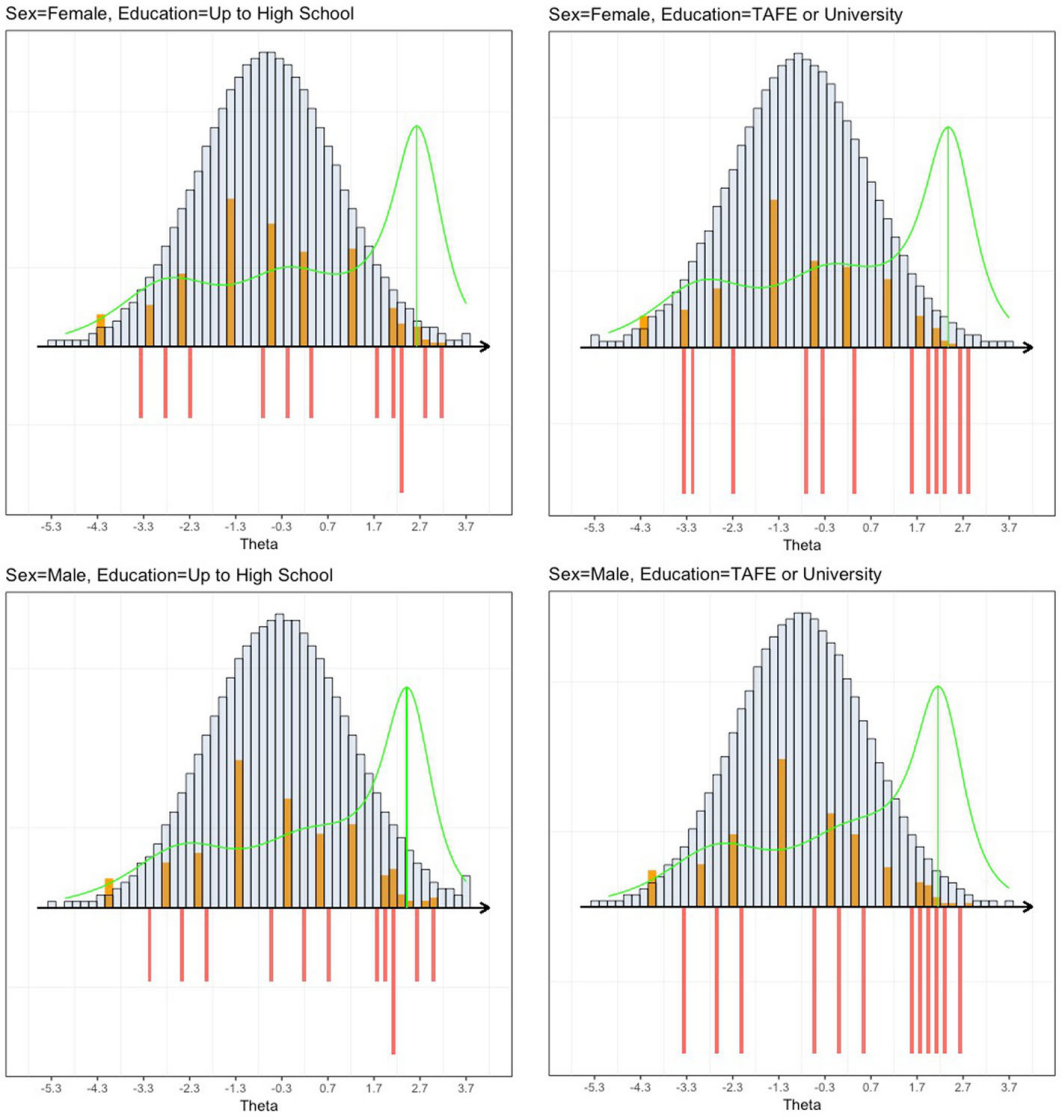
Item Map of the Perceived Control subscale according to subgroups. Note. The orange bars display the person parameters (weighted maximum likelihood estimates). The grey bars display the population distribution of Perceived Control under the assumption of normality. The red bars display the item thresholds and the green line is the information function

For the Perceived Control subscale, targeting was poor. The overall Test Information Target Index indicated that 34% of the total information was attained (Table 4) (Fig. 3). The overall reliability was 0.74 and the overall person separation probability was 75%.

### Criterion validity

The Perceived Stress and Perceived Control subscales displayed the expected patterns of convergence and divergence regarding the complementary measures (Supplementary Table 9 - Additional file 1). The analysis of known-groups validity indicated that women had higher scores of perceived stress (diff_adj_ = 0.67) but no substantial difference regarding perceived control (diff_adj_ = 0.04). Participants with education up to high school had lower scores on perceived control (diff_adj_ = − 0.50) but showed no substantial difference in perceived stress (diff_adj_ = 0.05) (Table 5).

**Table 5:** Observed and adjusted scores accounting for DIF

	Observed		Adjusted		Bias
	Mean	SE	Mean	SE	
Perceived Stress					
Education					
Up to High School	8.21	0.11	7.94	0.12	0.26
Technical education or University	8.04	0.07	7.89	0.07	0.15
Sex					
Female	8.50	0.08	8.16	0.08	0.34
Male	7.43	0.10	7.49	0.10	−0.06
Perceived Control					
Education					
Up to High School	7.81	0.06	7.92	0.07	−0.11
Technical education or University	8.17	0.04	8.42	0.04	−0.25
Sex					
Female	8.01	0.04	8.27	0.06	−0.26
Male	8.13	0.06	8.23	0.06	−0.11

It is displayed the average score for each subgroup before and after adjustment for differential item functioning. The bias indicate the differences between observed and adjusted scores

## Discussion

The aim of the present study was to evaluate if the PSS-14 constitutes a valid and reliable instrument to measure perceived stress in Australia. The results indicate that: 1) the revised version of the Perceived Stress subscale displayed adequate psychometric properties and provides a measure of perceived stress; however, 2) the majority of psychometric problems centered around the Perceived Control subscale. The implications for future use of the Perceived Stress Scale in Australia are discussed.

### Dimensionality

The results from the present study indicated that the PSS-14 is not unidimensional but rather composed of two dimensions. The observed correlation between the Perceived Stress and Perceived Control subscales (*γ*= 0.527) was strong but weaker than expected under a unidimensional model. The conclusion towards two dimensions (rather than one) was based not only from the dimensionality analysis but also considering the theoretical background of the PSS (Lee, 2012). The interpretation is that, although the two constructs of perceived stress and perceived control are *correlated* – as they are expected to be, since according to Lazarus [8] events are perceived as stressful when there is a perception of insufficient control over the situation – these constructs are nonetheless *qualitatively distinct*.

### Perceived stress subscale

The Perceived Stress subscale displayed adequate psychometric properties after the deletion of two items. The problems found with Item 12 ( “… have you found yourself thinking about all the things you have to accomplish?”), which was excluded in the original validation conducted by Cohen [12], have been extensively reported [33, 83–88]. It has been shown, for example, that Item 12 was endorsed by respondents with low *and* high levels of perceived stress, since “thinking about all the things you have to accomplish” does not necessarily mean being overwhelmed by them but also constitutes a self-management behaviour [87]. Studies that reported problems with Item 8 were less common [5, 89]. Finally, the Perceived Stress subscale displayed the expected pattern of convergent/divergent validity and known-groups validity except for education, providing further support for construct validity in the Australian population.

### DIF and gender bias

The findings of the current study were also consistent with the recent PSS literature regarding DIF. When DIF was investigated in relation to sex, DIF was found for Item 1 [47, 90], Item 3 [45, 47, 90], Item 6 [47] and Item 10 [45], similarly to previous studies. Rather than a characteristic specific to Australian respondents, the DIF of these items seems to be a consequence of *gender roles* present in Western societies, as documented by a robust body of psychological literature [91–93]. The traditional female gender role prescribes emotional expressiveness and lack of assertiveness, while the traditional male role prescribes assertiveness and self-confidence [94]. Matud [94] explains that “The stress associated with gender role identification is different for each sex because women are more likely to identify with the feminine gender role, and men are more likely to identify with the masculine gender role”. This is known as the *socialization hypothesis* [95] and the influence of gender roles on item response patterns has been previously reported in stress research. For example, Smith and Reise [96] showed that, compared to men with the same level of stress, women more frequently endorse items regarding emotional vulnerability and sensitivity.

In the present study, this DIF pattern was found in Item 1 ( “… felt upset because of something that happened unexpectedly?”) (γ = 0.24) and Item 3 ( “… felt either nervous or stressed?”) (γ = 0.33), which were more frequently endorsed by women. An opposite pattern was found in Item 6 (“...felt confident about your ability to handle your personal problems?”) (γ = − 0.15) and Item 10 ( “… felt you were on top of things?”) (γ = − 0.23), which were systematically endorsed by men. One possible explanation for these phenomena is that masculinity stereotypes in Western societies emphasize success, competition and *being in control*. Therefore, one possible explanation is that gender roles influenced response patterns and men were less likely to acknowledge negative emotions [97] and more likely to acknowledge self-confidence [94]. The pressure to hide vulnerabilities leads to underreporting of psychological symptoms among men and long-term consequences are under diagnosis and under treatment, creating a “silent epidemic” of mental illness [98, 99].

One main contribution of the present study is to provide evidence to the long-standing debate of “gender-related differences in PSS scores” [14]. The results demonstrated that women had higher levels of perceived stress even after scores were adjusted for measurement bias (diff_adj_ = 0.67; diff_obs_ = 1.07), since bias was responsible for 37% of the difference. Therefore, the differences of perceived stress scores between men and women in Australia is not explained by measurement bias alone and can be interpreted as true differences arising from social, biological and psychological influences [33]. However, it is necessary for future studies to investigate the impact of these differences. For example, the impact generated by a 0.67 higher average score in terms of use of the health system, psychopathology, disability leave, among others.

When DIF was analysed with respected to education, DIF was found for Item 1 and Item 10 ( “… felt you were on top of things?”). This result is congruent with Cole [45], who also showed that, given the same level of perceived control, participants with higher education were more likely to believe they *were on top of things*. Recent findings have suggested that perceived control is affected by educational attainment and is a mediator of health behaviours. For example, individuals with more educational attainment had a stronger belief that their actions would produce desirable outcomes (e.g. exercise and dieting would prevent developing disease) and had less fatalism. Additionally, feeling *on top of things* might also be interpreted as the relationship between higher education and status in western societies.

Since DIF was present among many of the PSS-14 items, a fundamental recommendation of the present study is that future applications of the Perceived Stress Scale in Australia need to use the conversion table (Supplementary Table 7 - Additional file 1) to adjust total scores and avoid measurement bias. The presence of DIF is a threat to construct validity since observed scores cannot be interpreted as reflecting true differences of perceived stress/perceived control. Therefore, if total scores are used without adjustment, the comparisons between subgroups are invalid.

### Response dependence

The present study showed positive LD between Item 1 ( “… felt upset because of something that happened unexpectedly?”) and Item 2 ( “… felt unable to control the important things in your life?”) (γ_avg_ = 0.18), and between Item 7 ( “… felt things were going your way?”) and Item 10 ( “… felt you were on top of things?”) (γ_avg_ = 0.22). The dependence between Item 1 and 2 [50], and between Item 7 and 10 [50, 90] have been previously reported; while the dependence between Item 7 and Item 10 found in Australia (γ_avg_ = 0.22) was also found in Danish students with a similar magnitude (γ_avg_ = 0.24) [90]. In these two pairs of items, the dependence seems to be a case of *response dependence* [100, 101]. For example, given the same trait level, respondents who endorsed Item 7 (“… felt things were going your way?”) had a higher probability of endorsing Item 10 (“… felt you were on top of things?”) than those who did not endorse the former. This seems to happen because *feeling on top of the things* in most cases logically imply that *things were going your way*.

### Problems with the perceived control subscale

The majority of psychometric problems were found on the Perceived Control subscale. Problems with the excluded Item 4 ( “… dealt successfully with irritating life hassles?”), Item 5 (“...effectively coped with important changes in your life?”) and Item 13 ( “… felt able to control the way you spend your time?”) have been reported by many [102–105]. Therefore, in conjunction with Item 12 from the Perceived Stress subscale, the exclusion of these three items indicate that the four items that were removed in the original validation by Cohen [12] that led to the creation of the PSS-10 once again performed poorly in Australia. For this reason, the application of the original PSS-14 in Australia is not warranted.

Furthermore, with the additional exclusion of Item 9 ( “… felt able to control irritations in your life?”), there are two implications for future studies. Firstly, the Perceived Control subscale was initially developed to be a *seven-item measure* of perceived coping/control through the theoretical perspective of Lazarus [8]. However, with the majority of items performing poorly, it seems unclear whether the three remaining items are enough to cover this psychological construct and poses concerns regarding *construct underrepresentation* [62]. Secondly, a subscale composed of three items might have reduced reliability, as happened in the current study, in which the overall reliability of the Perceived Control subscale was only moderate (*R* = 0.74) [106]. Therefore, the findings of this study suggest that: a) new items should be developed for the Perceived Control subscale to ensure construct validity for an Australian population; and b) if the 3-item Perceived Control subscale is applied, the results should be interpreted with caution.

### Theoretical constributions and limitations

The current study provides theoretical contributions to the validity of the PSS and to stress measurement. This study confirms the well-established findings regarding the two-dimensional structure of the PSS (“Perceived Stress” and “Perceived Control” subscales) and the preference towards the PSS-10 over the PSS-14 version due to 4 misfitting items. The two-dimensional structure indicates that total scores need to be computed for the “Perceived Stress” and “Perceived Control” subscales independently, instead of a total score summing across all items.

We also confirmed recent findings of DIF by gender of items 1 and 3, more easily endorsed by women, and items 6 and 10, more easily endorsed by men. We hypothesize that this DIF *pattern* is a consequence of gender roles present in Western societies, a response pattern similar to what has been reported in other stress measures [96]. We contribute to stress measurement by investigating whether score differences represent true gender differences or are solely a consequence of measurement bias. We showed that, although there is measurement bias due to DIF, this bias accounted for only 37% of score differences and the remaining difference on stress levels between men and women are real. A practical implication of this finding is that, due to measurement bias, scores need to be adjusted (using the conversion table) to enable an unbiased comparison of stress between Australian men and women.

This study also advances the literature of the PSS validity by investigating local dependence and targeting. We revealed that items 1 and 2, and 7 and 10 showed patterns of positive local dependence and that, if not taken into account, the dependence can lead to inflated estimates of reliability. Furthermore, we showed that the PSS is poorly targeted for a general high-income country population and is possibly better targeted for groups at risk of stress, such as students [48]. Future studies should also investigate the targeting of other stress measures. Targeting can become a bigger issue when, compared to our study, the instrument is applied to smaller samples from the Australian general population, leading to decreased reliability. It is possible that other stress measures are better targeted for the general population and should potentially be chosen over the PSS when evaluating stress in Australia at a national level.

One limitation of the present study is that the data available was from a national study conducted from 2004 to 2006. Considering that stress levels have increased over the years [2], the difference in the population distribution limits the *norm referenced* use of test scores [107]. That is, the use of the current sample as a *normative sample* should be used with caution, since the sample stress distribution does not correspond to the current population stress distribution in Australia. Nonetheless, the changes in the *stress distribution* of the Australia population by no means indicate that the PSS *item parameters* would also have changed. For instance, there are many psychological instruments, such as the Household Food Security Survey Module, which psychometric properties remained stable over decades [108]. Future longitudinal studies should consider administering again the PSS at a national level to investigate whether item parameters are stable over time (have longitudinal invariance [109]) or whether the measurement of stress is affected by item parameter drift (i.e. no longitudinal invariance).

Finally, the distribution of individual characteristics (such as sex, education, employment) in our large national sample was not representative of the distribution in the Australian population. While representativeness can sometimes be considered desirable, for instance when the study aim is primarily descriptive (e.g. describing the *prevalence* of stress in the general population), a non-representative sample does not entail that parameters (e.g. item difficulties) are biased [110] or impede the generalizability of the results [111]. The NSAOH 2004–2006 provided, to date, the best available evidence regarding the PSS-14 validity in the general Australian population.

## Conclusion

Research over half a decade has suggested high levels of stress in Australia, leading to critical consequences such as increased use of alcohol, cigarettes, and gambling as coping mechanisms. The present research showed that the Perceived Stress subscale is a valid and reliable measure of perceived stress after the deletion of two items. The majority of psychometric problems centered on the Perceived Control subscale. After the exclusion of four items, it is encouraged that new items should be developed to ensure construct representation or, if the short-form scale is applied, results should be interpreted with caution. Finally, a fundamental recommendation is that future applications need to use the conversion table to adjust total scores for measurement bias. If total scores are used without adjustment, the comparisons between population groups in Australia are invalid.

## Supplementary information

**Additional file 1: Table S1.** The PSS-14 items divided into Perceived Stress and Perceived Control subscales. **Table S2.** Item fit statistics for the PSS-14. **Table S3.** Item fit statistics for the Perceived Stress subscale. **Table S4.** Local dependence of the revised PSS-14 items. **Table S5.** Kelderman’s likelihood ratio tests for the GLLRM of Perceived Stress subscale. **Table S6.** Item fit statistics for the Perceived Control subscale. **Table S7.** Item fit statistics for the GLLRM of the Perceived Control subscale. **Table S8.** Kelderman’s likelihood ratio tests for the GLLRM of the Perceived Control subscale. **Table S9.** Conversion table for score adjustment. **Table S10**. Convergent and divergent validity of the PSS-14.

## Data Availability

The datasets generated and/or analysed during the current study are not publicly available since we do not have permission from the ethics committee to publicly release the datasets of the NSAOH 2004–2006 in either identifiable or de-identified form. The datasets are available from the corresponding author on reasonable request.

## Abbreviations

ABTSI: Aboriginal Australians and Torres Strait Islanders
APS: Australian Psychological Society
CLR: Conditional Likelihood Ratio
DIF: Differential Item Functioning
EF: Effort
ERI: Efforts-Reward Imbalance Questionnaire
FA: Family
FDR: False discovery rate
FR: Friends
GLLRM: Graphical Loglinear Rasch Model
IRT: Item Response Theory
LD: Local dependence
MSPSS: Multidimensional Scale of Perceived Social Support
NSAOH: National Survey of Adult Oral Health
OC: Over commitment
PSS: Perceived Stress Scale
RD: Reward
RM: Rasch Model
SO: Significant others
SWBS: Stress & Well-Being Survey
US: United States
WML: Weighted maximum likelihood

## Footnotes

1 Technical and Further Education (or TAFE) is the biggest provider of post-secondary education in Australia. TAFE offers a broad range of courses, at the operative, trade and paraprofessional level, that can last from a few hours (refreshment courses) to three years (diploma courses). Unlike universities, which are composed mostly of full-time students, TAFE institutions allow students to combine study and work, and encourage programs of apprenticeships and traineeships [73].

2 For simplicity, the term uniform is omitted when referred to uniform LD or uniform DIF from now on.
