# Supplementary material for: Measuring stress in Australia: validation of the perceived stress scale (PSS-14) in a national sample
Source: Health Qual Life Outcomes. 2020 Apr 15;18:100. doi: 10.1186/s12955-020-01343-x (PMC7161288; doi:10.1186/s12955-020-01343-x)
Supplement: Supplementary file 1 — Additional file 1: Table S1. The PSS-14 items divided into Perceived Stress and Perceived Control subscales. Table S2. Item fit statistics for the PSS-14. Table S3. Item fit statistics for the Perceived Stress subscale. Table S4. Local dependence of the revised PSS-14 items. Table S5. Kelderman’s likelihood ratio tests for the GLLRM of Perceived Stress subscale. Table S6. Item fit statistics for the Perceived Control subscale. Table S7. Item fit statistics for the GLLRM of the Perceived Control subscale. Table S8. Kelderman’s likelihood ratio tests for the GLLRM of the Perceived Control subscale. Table S9. Conversion table for score adjustment. Table S10. Convergent and divergent validity of the PSS-14. [file 12955_2020_1343_MOESM1_ESM.docx]

Table S1

*The PSS-14 items divided into Perceived Stress and Perceived Control subscales*

| Item number | Item content |
| --- | --- |
|  | Perceived Stress (PS) subscale |
| 1 | … felt upset because of something that happened unexpectedly? |
| 2 | … felt unable to control the important things in your life? |
| 3 | … felt either nervous or stressed? |
| 8 | … felt unable to cope with all the things that you had to do? |
| 11 | … felt angered because of things that happened outside of your control? |
| 12 | … found yourself thinking about all the things that you have to accomplish? |
| 14 | … felt difficulties were piling up so high that you could not overcome them? |
|  | Perceived Control (PC) subscale |
| 4 | … dealt successfully with irritating life hassles? |
| 5 | … effectively coped with important changes in your life? |
| 6 | … felt confident about your ability to handle your personal problems? |
| 7 | … felt things were going your way? |
| 9 | … felt able to control irritations in your life? |
| 10 | … felt you were on top of things? |
| 13 | … felt able to control the way you spend your time? |

*Note.* Every item started with the sentence “How often during the last year have you…”

Table S2

*Item fit statistics for the PSS-14*

|  | Conditional Outfit | | | Conditional Infit | | | Item-Restscore correlation | | |
| --- | --- | --- | --- | --- | --- | --- | --- | --- | --- |
| Item | Observed | SE | *p*-value | Observed | SE | *p*-value | Observed | Expected | *p-*value |
| Item 4 | 1.556 | 0.024 | <0.001 | 1.449 | 0.024 | <0.001 | 0.266 | 0.471 | <0.001 |
| Item 5 | 1.550 | 0.024 | <0.001 | 1.404 | 0.024 | <0.001 | 0.319 | 0.477 | <0.001 |
| Item 6 | 0.808 | 0.024 | <0.001 | 0.810 | 0.025 | <0.001 | 0.657 | 0.468 | <0.001 |
| Item 7 | 0.818 | 0.023 | <0.001 | 0.805 | 0.023 | <0.001 | 0.624 | 0.466 | <0.001 |
| Item 9 | 1.493 | 0.023 | <0.001 | 1.329 | 0.023 | <0.001 | 0.399 | 0.483 | <0.001 |
| Item 10 | 0.661 | 0.024 | <0.001 | 0.660 | 0.024 | <0.001 | 0.747 | 0.471 | <0.001 |
| Item 13 | 1.181 | 0.023 | <0.001 | 1.134 | 0.023 | <0.001 | 0.444 | 0.481 | <0.001 |
| Item 1 | 0.959 | 0.024 | 0.090 | 0.958 | 0.024 | 0.080 | 0.525 | 0.478 | <0.001 |
| Item 2 | 0.731 | 0.023 | <0.001 | 0.727 | 0.023 | <0.001 | 0.657 | 0.489 | <0.001 |
| Item 3 | 0.820 | 0.023 | <0.001 | 0.822 | 0.023 | <0.001 | 0.616 | 0.488 | <0.001 |
| Item 8 | 0.887 | 0.023 | <0.001 | 0.876 | 0.023 | <0.001 | 0.592 | 0.477 | <0.001 |
| Item 11 | 0.951 | 0.023 | 0.036 | 0.949 | 0.024 | 0.029 | 0.521 | 0.475 | <0.001 |
| Item 12 | 1.452 | 0.022 | <0.001 | 1.437 | 0.022 | <0.001 | 0.257 | 0.481 | <0.001 |
| Item 14 | 0.726 | 0.023 | <0.001 | 0.728 | 0.024 | <0.001 | 0.657 | 0.478 | <0.001 |

*Note.* The Conditional Outfit and Conditional Infit statistics have expected values equal to one under the Rasch model. The Item-Restscore correlation compares the observed item-restscore correlation with the expected item-restscore correlation under the model.

Table S3

*Item fit statistics for the Perceived Stress subscale*

|  | Conditional Outfit | | | | Conditional Infit | | | | Item-Restscore correlation | | |
| --- | --- | --- | --- | --- | --- | --- | --- | --- | --- | --- | --- |
| Item | | Observed | SE | *p*-value | | Observed | SE | *p*-value | Observed | Expected | *p-*value |
| Item 1 | | 0.942 | 0.024 | 0.016 | | 0.946 | 0.023 | 0.021 | 0.653 | 0.618 | 0.001 |
| Item 2 | | 0.750 | 0.023 | <0.001 | | 0.751 | 0.023 | <0.001 | 0.739 | 0.622 | <0.001 |
| Item 3 | | 0.801 | 0.024 | <0.001 | | 0.805 | 0.023 | <0.001 | 0.717 | 0.624 | <0.001 |
| Item 8 | | 1.007 | 0.023 | 0.748 | | 1.003 | 0.023 | 0.904 | 0.654 | 0.615 | <0.001 |
| Item 11 | | 1.010 | 0.023 | 0.659 | | 1.020 | 0.023 | 0.398 | 0.617 | 0.615 | 0.821 |
| Item 12 | | 1.675 | 0.023 | <0.001 | | 1.669 | 0.023 | <0.001 | 0.367 | 0.617 | <0.001 |
| Item 14 | | 0.804 | 0.023 | <0.001 | | 0.813 | 0.023 | <0.001 | 0.714 | 0.615 | <0.001 |

*Note.* The Conditional Outfit and Conditional Infit statistics have expected values equal to one under the Rasch model. The Item-Restscore correlation compares the observed item-restscore correlation with the expected item-restscore correlation under the model.

Table S4

*Local dependence of the revised PSS-14 items*

| Item pair | Partial γ |
| --- | --- |
| Item 1– Item 2 | 0.18 |
| Item 1 – Item 11 | -0.23 |
| Item 2 – Item 3 | 0.05 |
| Item 3 – Item 14 | 0.03 |
| Item 6 – Item 10 | 0.05 |
| Item 7 – Item 10 | 0.22 |

*Note.* Average partial gamma given the items’ restscores.

Table S5

*Kelderman’s likelihood ratio tests for the GLLRM of Perceived Stress subscale*

| Items | Conditional Likelihood Ratio test | | | Obs $\gamma$ |
| --- | --- | --- | --- | --- |
|  | Differential Item Functioning | | |  |
| Item 2 & Education: | lr =   12.23 | df =   4 | p = 0.016 | 0.11 |
| Item 3 & Education: | lr =    3.35 | df =   4 | p = 0.501 |  |
| Item 11 & Education: | lr =    3.98 | df =   4 | p = 0.408 |  |
| Item 14 & Education: | lr =    3.97 | df =   4 | p = 0.410 |  |
| Item 2 & Sex: | lr =    3.93 | df =   4 | p = 0.416 |  |
| Item 11 & Sex: | lr =    6.66 | df =   4 | p = 0.155 |  |
| Item 14 & Sex: | lr =    6.80 | df =   4 | p = 0.147 |  |
|  |  |  |  |  |
|  | Local Dependence | | | |
| Item 1 & Item 3: | lr =   81.60 | df = 16 | p <0.001 | 0.11  0.08 |
| Item 1 & Item 14: | lr =   52.69 | df = 16 | p <0.001 | -0.31 -0.26 |
| Item 2 & Item 11: | lr =   96.56 | df = 16 | p <0.001 | -0.23 -0.07 |
| Item 11 & Item 14: | lr =   91.18 | df = 16 | p <0.001 | 0.03  0.18 |
| Item 3 & Item 11: | lr =   92.19 | df = 16 | p <0.001 | -0.14 -0.03 |
| Item 11 & Item 14: | lr =   56.11 | df = 16 | p <0.001 | 0.05  0.02 |

*Note.* After the Benjamini-Hochberg procedure, statistical significance was adjusted as *p* < 0.02692 for a 5% FDR.

Table S6

*Item fit statistics for the Perceived Control subscale*

|  | Conditional Outfit | | | | Conditional Infit | | | | Item-Restscore correlation | | |
| --- | --- | --- | --- | --- | --- | --- | --- | --- | --- | --- | --- |
| Item | | Observed | SE | *p*-value | | Observed | SE | *p*-value | Observed | Expected | *p-*value |
| Item 4 | | 1.201 | 0.024 | <0.001 | | 1.134 | 0.024 | <0.001 | 0.482 | 0.505 | 0.086 |
| Item 5 | | 1.152 | 0.024 | <0.001 | | 1.083 | 0.024 | <0.001 | 0.521 | 0.509 | 0.372 |
| Item 6 | | 0.680 | 0.025 | <0.001 | | 0.704 | 0.025 | <0.001 | 0.704 | 0.500 | <0.001 |
| Item 7 | | 0.930 | 0.023 | 0.003 | | 0.907 | 0.024 | <0.001 | 0.585 | 0.501 | <0.001 |
| Item 9 | | 1.367 | 0.023 | <0.001 | | 1.237 | 0.023 | <0.001 | 0.457 | 0.513 | <0.001 |
| Item 10 | | 0.763 | 0.024 | <0.001 | | 0.779 | 0.024 | <0.001 | 0.661 | 0.505 | <0.001 |
| Item 13 | | 1.222 | 0.023 | <0.001 | | 1.187 | 0.023 | <0.001 | 0.442 | 0.511 | <0.001 |

*Note.* The Conditional Outfit and Conditional Infit statistics have expected values equal to one under the Rasch model. The Item-Restscore correlation compares the observed item-restscore correlation with the expected item-restscore correlation under the model.

Table S7

*Item fit statistics for the GLLRM of the Perceived Control subscale*

|  | Conditional Outfit | | | | Conditional Infit | | | | Item-Restscore correlation | | |
| --- | --- | --- | --- | --- | --- | --- | --- | --- | --- | --- | --- |
| Item | | Observed | SE | *p*-value | | Observed | SE | *p*-value | Observed | Expected | *p-*value |
| Item 6 | | 0.979 | 0.033 | 0.532 | | 0.994 | 0.033 | 0.867 | 0.719 | 0.711 | 0.470 |
| Item 7 | | 0.981 | 0.033 | 0.572 | | 0.998 | 0.030 | 0.946 | 0.744 | 0.739 | 0.633 |
| Item 10 | | 1.022 | 0.042 | 0.594 | | 1.023 | 0.038 | 0.538 | 0.781 | 0.783 | 0.877 |

*Note.* The Conditional Outfit and Conditional Infit statistics have expected values equal to one under the Rasch model. The Item-Restscore correlation compares the observed item-restscore correlation with the expected item-restscore correlation under the model.

Table S8

*Kelderman’s likelihood ratio tests for the GLLRM of the Perceived Control subscale*

| Items | Conditional Likelihood Ratio test | | | Obs $\gamma$ |
| --- | --- | --- | --- | --- |
|  | Differential Item Functioning | | |  |
| Item 6 & Education: | lr =   12.59 | df =   4 | p = 0.014 |  |
| Item 7 & Education: | lr =    3.36 | df =   4 | p = 0.449 |  |
| Item 7 & Sex: | lr =    0.64 | df =   4 | p = 0.982 |  |
|  |  |  |  |  |
|  | Local Dependence | | | |
| Item 6 & Item 7: | lr =  128.04 | df = 16 | p<0.001 | -0.16 -0.28 |

*Note.* After the Benjamini-Hochberg procedure, statistical significance was adjusted as *p* < 0.005 for a 5% FDR.

Table S9

*Conversion table for score adjustment*

| Score | Adjusted Scores | | | |
| --- | --- | --- | --- | --- |
| Perceived Stress Subscale | Sex: Male  Education: Up to high school | Sex: Male  Education: TAFE or University | Sex: Female  Education: Up to high school | Sex: Female  Education: TAFE or University |
| 1 | 1.00 | 0.99 | 0.72 | 0.71 |
| 2 | 2.00 | 2.00 | 1.5 | 1.50 |
| 3 | 3.00 | 3.03 | 2.38 | 2.41 |
| 4 | 4.00 | 4.07 | 3.36 | 3.45 |
| 5 | 5.00 | 5.11 | 4.43 | 4.56 |
| 6 | 6.00 | 6.14 | 5.52 | 5.67 |
| 7 | 7.00 | 7.14 | 6.6 | 6.74 |
| 8 | 8.00 | 8.11 | 7.68 | 7.78 |
| 9 | 9.00 | 9.07 | 8.73 | 8.79 |
| 10 | 10.00 | 10.04 | 9.72 | 9.74 |
| 11 | 11.00 | 11.02 | 10.66 | 10.67 |
| 12 | 12.00 | 12.01 | 11.61 | 11.62 |
| 13 | 13.00 | 13.03 | 12.59 | 12.6 |
| 14 | 14.00 | 14.05 | 13.59 | 13.64 |
| 15 | 15.00 | 15.08 | 14.63 | 14.71 |
| 16 | 16.00 | 16.11 | 15.70 | 15.8 |
| 17 | 17.00 | 17.11 | 16.79 | 16.9 |
| 18 | 18.00 | 18.08 | 17.89 | 17.97 |
| 19 | 19.00 | 19.04 | 18.96 | 19 |
| Perceived Control subscale | Sex: Male  Education: Up to high school | Sex: Male  Education: TAFE or University | Sex: Female  Education: Up to high school | Sex: Female  Education: TAFE or University |
| 1 | 1.00 | 1.49 | 0.67 | 0.99 |
| 2 | 2.00 | 2.81 | 1.39 | 2.09 |
| 3 | 3.00 | 3.81 | 2.30 | 3.16 |
| 4 | 4.00 | 4.62 | 3.45 | 4.21 |
| 5 | 5.00 | 5.39 | 4.80 | 5.24 |
| 6 | 6.00 | 6.22 | 6.11 | 6.33 |
| 7 | 7.00 | 7.13 | 7.31 | 7.41 |
| 8 | 8.00 | 8.08 | 8.30 | 8.36 |
| 9 | 9.00 | 9.07 | 9.26 | 9.32 |
| 10 | 10.00 | 10.12 | 10.30 | 10.42 |
| 11 | 11.00 | 11.10 | 11.20 | 11.28 |

Note. The table indicates conversion values for comparison among subgroups. For example, if a woman with education up to high school had on the Perceived Stress subscale a observed total scores of 10, the score should be adjusted to the (true) value of 9.72.

Table S10

*Convergent and divergent validity of the PSS-14*

|  | FR | 95% CI | FA | 95% CI | SO | 95% CI | EF | 95% CI | RW | 95% CI |
| --- | --- | --- | --- | --- | --- | --- | --- | --- | --- | --- |
| Perceived Stress | -0.15 | [-0.20, -0.10] | -0.22 | [-0.27, -0.17] | -0.16 | [-0.21, -0.11] | 0.19 | [0.12, 0.25] | -0.26 | [-0.29, -0.16] |
| Perceived Control | 0.23 | [0.18, 0.28] | 0.25 | [0.30, 0.21] | 0.28 | [0.20, 0.29] | -0.09 | [-0.16, -0.03] | 0.28 | [0.22, 0.34] |

*Note.* MS = Mastery; PC = Perceived Constraints; SSS = Social Support Scale; FR = Friends; FA = Family; SO = Significant others; EF = Effort; RW = Rewards.
